# Transition from glass- to gel-like states in clay at a liquid interface

**DOI:** 10.1038/srep37239

**Published:** 2016-11-24

**Authors:** A. Gholamipour-Shirazi, M. S. Carvalho, M. F. G. Huila, K. Araki, P. Dommersnes, J. O. Fossum

**Affiliations:** 1 Department of Mechanical Engineering, Pontificia Universidade Catolica do Rio de Janeiro, Rio de Janeiro, RJ, Brazil; 2 Institute of Chemistry, Universidade de São Paulo - USP, Sao Paulo, SP, Brazil; 3 Department of Physics, Norwegian University of Science and Technology - NTNU, Trondheim, Norway

## Abstract

Colloidal clay in water suspensions are known to exhibit a multitude of bulk phases depending on initial colloidal concentration and ionic strength, and examples of this include repulsive Wigner colloidal glasses at low ionic strength and attractive gels at higher ionic strength due to screened electrostatic forces by the electrolyte. From confocal Raman microscopy combined with elasticity measurements, we infer that clay trapped at quasi two-dimensional interfaces between oil and water also exhibit confined glass-like or gel-like states. The results can be important for the preparation of particles stabilized colloidal emulsions or colloidal capsules, and a better understanding of this phenomenon may lead to new emulsion or encapsulation technologies.

Colloidal clays display very rich phase diagrams with a variety of interfering states, including disordered phases of fluids, gels and glasses^1^,^2^,^3^, as well as ordered nematic and columnar phases^4^,^5^. A well-studied model system in this context is Laponite^1^,^2^,^3^,^4^,^5^,^6^,^7^,^8^,^9^,^10^,^11^,^12^,^13^, which is a synthetic smectite clay composed of monodisperse 1 nanometer thick disks with 1:25 aspect ratio with negative surface charges, and small positive rim charges^1^,^2^,^3^,^4^,^5^,^6^,^7^,^8^,^9^,^10^,^11^,^12^,^13^. The net negative charge of smectite colloidal particles such as Laponite is compensated by cations. In addition, the surface charges can be effectively screened by adding salt to aqueous dispersions. Thus one can, in such systems, tune the sign and the magnitude of effective interactions between individual colloidal clay platelets by controlling the amount of salt in the water. Therefore, one particular feature of the Laponite phase diagram that has received interest from a fundamental point of view, is the salt controlled transition between glass (interparticles repulsion forming Wigner glasses) to gels (interparticles attraction forming networks)^2^,^3^,^11^,^12^,^13^. In bulk smectite clays, such salt and particle concentration controlled nanostructures are intimately linked to their macroscopic mechanical and flow behaviors^1^,^2^,^3^,^14^,^15^.

Particle-stabilized emulsions (i.e. Pickering emulsions)^16^,^17^ are receiving increasing attention due to their superior stability when compared to traditional emulsions stabilized by surfactant molecules^18^,^19^,^20^,^21^. Both Pickering emulsions and clays can be found in a wide range of industries such as food, pharmaceutical, paint and petrochemical, and there is thus both fundamental and practical interest in “combining the two” and study clay particle stabilized Pickering emulsions^22^,^23^. Previous works in this direction have studied clay based Pickering oil-in-water emulsion stability^23^, and mechanical properties of individual clay armored oil drops^24^,^25^. The motivation for the present work is to investigate whether salt and particle concentration controlled smectite clay nanostructures and their resulting mechanical behaviors also are present in Laponite Pickering films on oil drops in water, similar to the phenomena described above for bulk Laponite. This is linked to questions concerning the control of the mechanical and flow behavior of smectite clay Pickering films on drops, and ultimately the possibility for pre-designing the strength and stability of particle (in the present case smectite clay) stabilized Pickering emulsions.

Previous recent studies of mechanical properties of particle covered drops^24^,^26^,^27^ and liquid marble mechanical stability^28^ have employed methods including the pendant drop method^26^. In the present work, we combine confocal Raman microscopy^29^ and the oscillating pendant drop method^30^ to visualize and characterize Pickering interfaces, as well as to quantify their viscous and elastic moduli. We demonstrate that like for the bulk case, the transition from a glass- to a gel-like state of the clay particles at the liquid interface can be controlled by the clay concentration and the ionic strength of the electrolyte^1^,^2^,^3^,^31^.

## Results

In the present work we have used Laponite RD^®^ synthetic clay colloidal suspensions as the aqueous phase with varying concentrations of clay particles (from 0% to 1.5% by weight) and different salinities (0 and 0.1 M of NaCl). All clay suspensions were prepared in concentrations, and observed to be, outside the gelation region on the phase diagram^1^. The oil phase was a purified paraffinic mineral oil (Drakeol7^®^). Our investigations of the produced oil water interfaces demonstrate a glass-like or a gel-like state of the Laponite self-organization, which is significantly dependent on ionic strength.

Confocal Raman microscopy is a powerful tool for chemical imaging of materials especially in aqueous phase, due to the low scattering cross-section of this solvent. In fact, it is possible to image with lateral resolution as high as 150 nm as can be confirmed by the example shown in Supplementary Figure 1 where a 607-STM Waffle Grating Replica for STM calibration from Ted Pella^®^ was imaged. The crossing point and squares defined by lines separated by 460 nm can be clearly seen in the Raman image (Supplementary Figure 1a) as bright spots whose size is dependent on the chosen intensity threshold, as confirmed by the on line and diagonal cross-section profiles shown in Supplementary Figure 1b.

**Figure 1:**
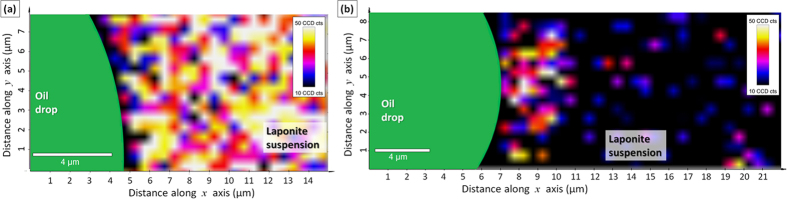
Confocal Raman microscopy of a single oil drop (**a**) in Laponite 1.5 wt% dispersion in DI-water; and (**b**) in Laponite 0.75 wt% dispersion in 0.1 M NaCl obtained by dilution of Laponite 1.5 wt% with the same salt solution (more details in Supplementary Figure 4b). The pictures display a two-dimensional Raman mapping of the oil (green area to the left), Laponite tactoids (yellow/red/white) and water (black) positions. The 2–4 μm thick film of nanostructured Laponite tactoids adsorbed at the oil water interface cannot be seen unless the aqueous phase is diluted with NaCl solution as shown experimentally in Fig. 2c.

Accordingly, confocal Raman microscopy was used to reveal the Pickering interfacial films, and images scanning a horizontal plane of 13 × 10 μm^2^ crossing the interface between an oil drop dispersed in different water phases were obtained, as displayed in Fig. 1, revealing the presence not of individual clay platelets (laponite is constituted by disc shaped about 30 nm diameter and 1 nm thick nanoparticles) but rather of tactoids, aggregated laponite particles. For detailed investigations, normalized linearly scanned concentration profiles of each phase near and across the oil-water interface were obtained before and after diluting the water phase and consequently reducing the bulk Laponite concentration by 50%, from 1.5 to 0.75 wt.%. We monitored the main Raman peaks in the 630 to 1710 cm^−1^ range for Laponite, oil and water, appearing at 680, 1445 and 1630 cm^−1^ respectively, corresponding to symmetric Si-O-Si stretching in laponite, CH_2_ bending in mineral oil and HOH bending in water (Supplementary Figures 2 and 3). Clearly, the Laponite tactoids are more or less homogeneously dispersed in pure water arranged in structures that resemble a house of cards, and has a tendency to avoid the oil/water interface, as confirmed by the dark interface indicating the presence of a water layer. At this point, it is important to stress that no significant change was observed upon dilution of suspension with pure water. In contrast, when salt was added into the aqueous phase and the emulsion diluted, a nanostructured layer of Laponite tactoids remained strongly adsorbed at the oil/water interface generating a stable Pickering shell.

**Figure 2:**
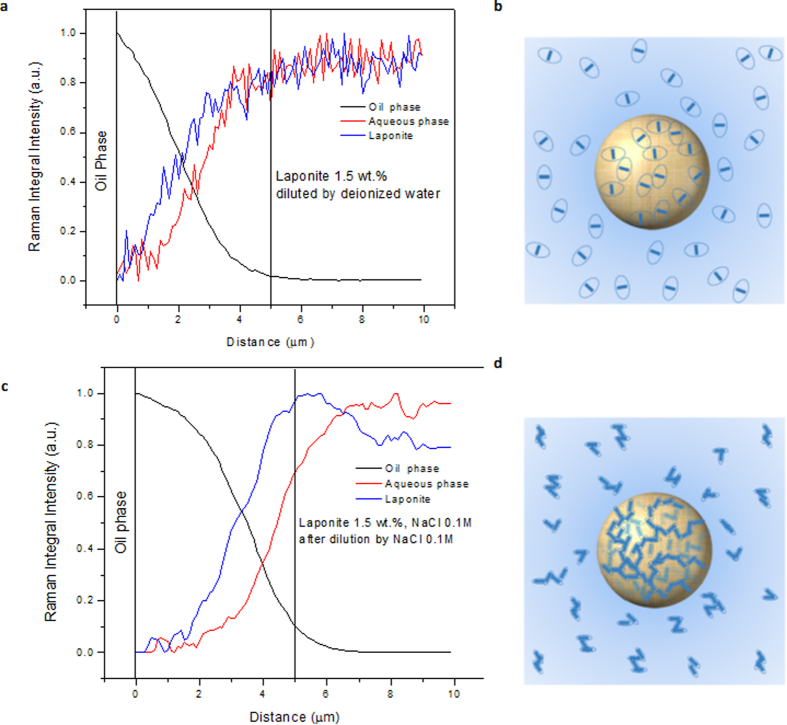
The effect of salt on Laponite nanoparticles structure at the interface. Concentration profiles across the interface after dilution of the water phase for (**a**) system without salt and (**c**) system with salt (0.1 M NaCl). Figures b and d show proposed schematic (not to scale) representation of the particle structure for the system without and with salt, respectively. For the system without salt (**b**), a repulsive “Wigner” colloidal glass is formed at the interface. For the system with salt (**d**), a particle network is formed at the interface, leading to a gel state. In the schematic representation, each thick line represents a Laponite disk, while the ellipsoids around them represent the range of electrostatic repulsions.

The result for the diluted system with no salinity (Debye screening length, 311 nm^32^) is shown in Fig. 2a. The Laponite concentration in the interface is lower than in the bulk phase. The dilution of the water phase did not affect the relative distribution of clay particles between the bulk and the interface (the non-normalized intensity of Laponite peak across the interface for the diluted and original Laponite concentration in the water phase are shown in Supplementary Figure 4d,c). Based on the images shown in Fig. 1a, we propose that without salt, the Laponite distribution in the bulk and interface could be represented as sketched in Fig. 2b. Long-range electrostatic repulsions dominate and so the Laponite nanoparticles state is “Wigner” colloidal glass^33^. The behaviour of the system with salt (0.1 M NaCl) is completely different. The concentration profile before the dilution step is similar to that of the system without salt (see Supplementary Figure 4a). However, after dilution, the Laponite signal at the interface becomes stronger than that of the bulk, as shown in Fig. 2c and Supplementary Figure 4b. Together with the interconnected Laponite signal distributed through the water phase in the interface region shown in Fig. 1b, this is strong evidence that Laponite particles are trapped at the interface and that a particle network is formed forming a stabilizing layer of Laponite with the thickness of about 4 μm. Attractive interactions play a dominant role, a percolated network forms, as sketched in Fig. 2d, which gives the system its elasticity^33^.

In order to characterize the interface mechanical behaviour of the Laponite based Pickering films, the pendant drop method^30^,^34^,^35^,^36^ was used. The equilibrium shapes of the oil drop in the 1.5 wt.% Laponite, 0.1 M NaCl dispersion for a short (few seconds) and for a very long (>10 hours) aging time are shown in Supplementary Figure 5a,b respectively. The configuration of the interface for long aging time does not follow the “Laplacian” shape observed in the fresh formed interface and approaches a spherical shape. Subsequently, for the long time aged drop, the volume was gradually reduced by pulling the oil phase back into the syringe. Supplementary Figure 5c shows the resulting interface shape, where a crumpling behavior is observed, which is evidence of a solid like, irreversible film formation at the interface.

**Figure 3:**
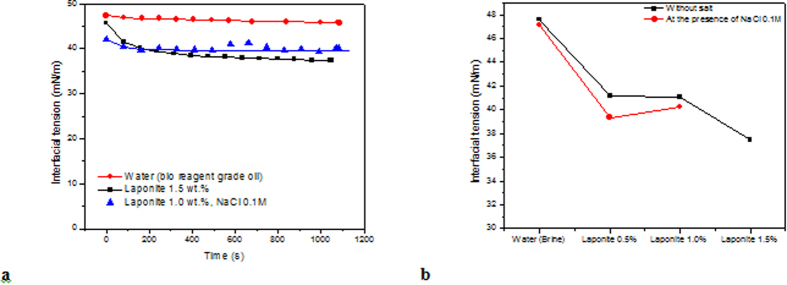
(**a**) Time evolution of Laponite dispersions interfacial tension. (**b**) Steady state values of interfacial tension for different Laponite suspensions.

The evolution of the interfacial tension with time, measured in a constant drop volume experiment, for all the investigated Laponite dispersions is shown in Fig. 3a. For all cases, the interfacial tension falls slowly with time before asymptotically reaching a steady plateau value. The steady state value of interfacial tension for the different suspensions is presented in Fig. 3b.

The addition of Laponite nanoparticles to the interface lowers the interfacial tension. However, the effect of Laponite concentration on the steady-state interfacial tension is not strong; it varies from 48 mN/m for the pure water case to 38 mN/m for the 1.5% Laponite suspension. Adding the salt does not affect significantly the steady state value of the interfacial tension.

The relationship between the applied strain (area deformation) and resulting stress (interfacial traction) is the basis for evaluating interfacial dilational rheology behavior^30^. After the initial transient decay, the periodic oscillation of the surface area leads to a sinusoidal variation of the surface traction. This behavior can be characterized by the dilational modulus *ε*, defined as the interfacial traction variation per unit of fractional change in the interfacial area (A):


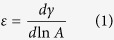


The dilational phase shift, *φ*, is equal to 0 for purely elastic and to π/2 for purely viscous interfaces^30^. The relaxation processes at or near the interface between water and oil lead to a viscoelastic behavior of the interface film, for which the phase shift angle is between 0 and π/2. The oscillatory response is characterized by the dynamic dilational modulus which is a complex quantity. Its real and imaginary parts correspond to the elastic and viscous contributions *ε* = *ε*_*d*_ + *iωη*_*d*_, where *ε*_*d*_, *ω* and *η*_*d*_ are the dilational interfacial elastic modulus, the oscillation frequency and the interfacial dilational viscosity, respectively. The magnitude of the modulus |*ε*| is simply the square root of the sum of the elastic and viscous components^30^. Freer *et al*.^36^ have shown that viscous forces distort the drop shape at high capillary number, e.g. Ca > 0.002. In our experiments, the capillary number was in the range of 4.0 × 10^−5^ to 1.65 × 10^−4^, which implies that the viscous forces do not distort the drop shape and that its configuration is mainly dictated by forces acting on the interface, not coming from the bulk of any of the phases. Moreover, the Laponite and salt concentration were such that the suspensions were outside the gelation region of the bulk phase diagram. Without salt and 1.5% Laponite, we observed that the suspension behaves like an isotropic liquid at the timescales investigate here; whereas at 0.1 M NaCl and 1.5% Laponite, the suspension is in the Flocculation regime^1^ and does not show any viscoelastic behavior. Therefore, any time dependent response observed in the oscillatory tests is not related to the bulk behavior and can be directly associated with the interfacial behavior.

For pure interfaces, the surface traction should not depend on the surface area and the dilational modulus should be equal to zero. The evolution of the interfacial traction as a function of time for an imposed periodic oscillation of the surface area for the pure water and the 1.5% Laponite and 0.1 M NaCl suspension is presented in Supplementary Figure 6. For the pure water case, a small variation of surface traction with interfacial area was observed, probably due to the impurities in the oil phase (Supplementary Figure 6a). For viscoelastic interfaces, the change in surface area and change in surface tension are out of phase (Supplementary Figure 6b). The value of the interfacial tension, i.e. the average of the interface traction measured in the oscillatory tests, for most of the suspensions tested (different Laponite and salt concentration) were similar to those obtained in the constant interfacial area experiments. The results for different oscillation frequency are shown in Supplementary Figures 7 (f = 0.2 Hz) and 8 (f = 0.1 Hz). At higher Laponite and NaCl concentrations, an increase in the error associated with the measurement is observed, because as discussed later, in this case, clay particles will form a structure in the interface, leading to a strong elastic response, and the obtained profile is not adequately fitted by the Young-Laplace profile.

As observed in Supplementary Figures 7 and 8, the interfacial tension slightly falls by increasing Laponite concentration for zero salt samples. Reger *et al*.^37^ have shown that soluble Laponite XLG–amphiphile complexes have a weak effect on the surface tension of water. In the present case, a small decrease in interfacial tension after adding the electrolyte to the Laponite 0.5 wt.% dispersion is also. Verruto *et al*.^38^ observed that the interfacial tension of an asphaltenic film at the oil-water interface is decreased with salt addition both at acidic (pH 3) and basic (pH 10) aqueous phases. This could be attributed to limiting the interfacial packing and, hence, the magnitude of the interfacial tension by repulsive electrostatic interactions at low ionic strength. By adding the electrolyte, the Coulomb repulsion is screened; the asphaltenic aggregates can pack more densely on the interface, and the interfacial tension is reduced^38^. Similar phenomena are active in a number of systems^39^, including in the case of Laponite interfacial film assemblies. At higher Laponite concentration, the addition of electrolyte raises the interfacial tension. This can be associated with the formation of the particle network at the interface.

**Figure 4:**
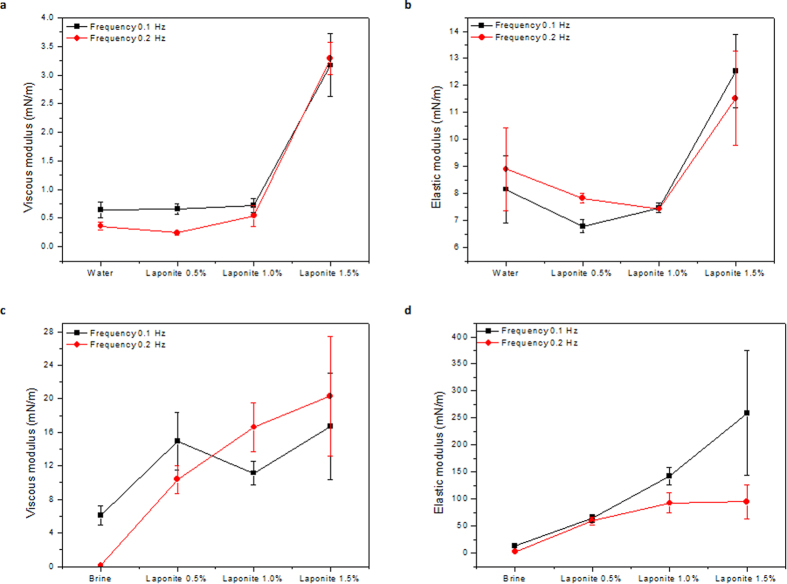
The interfacial viscoelastic moduli for Laponite dispersions (**a**) Viscous modulus and (**b**) Elastic modulus in the absence of salt, (**c**) Viscous modulus and (**d**) Elastic modulus in the presence of salt.

The interfacial viscous and elastic dilational moduli measured at two different frequencies for all the suspensions without and with salt are shown in Fig. 4a–d.

In the salt free dispersions, the viscous and elastic moduli of Laponite 0.5 and 1 wt.% are very low and almost equal to the pure water interface. The moduli of Laponite 1.5 wt.% are higher, but still low and much smaller than interfacial tension. Ashby and Binks^40^ have shown that salt free Laponite dispersions do not lead to stable emulsions, indicating that the interface properties in this case are similar to pure water-oil interface. We observe that the mechanical response for salt free dispersions is independent of the imposed frequency. After adding salt, a significant increase is observed for both the viscous and elastic modulus. However, still the value of viscous modulus is negligible as compared to the elastic modulus value, which is approximately 250 mN/m for the 1.5% Laponite suspension at f = 0.1 Hz (f is oscillation frequency).

**Figure 5:**
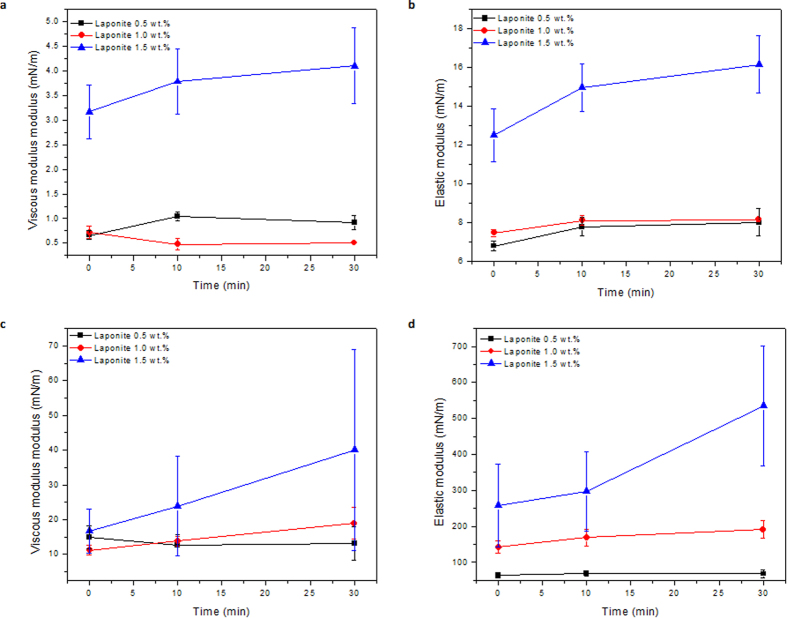
The effect of aging time on the interfacial viscoelastic moduli of different Laponite dispersions. Volume amplitude ratio for all cases is 2 μL and the frequency is 0.1 Hz. (**a**) Viscous modulus and (**b**) Elastic modulus in the absence of salt. (**c**) Viscous modulus at and (**d**) Elastic modulus in the presence of salt.

The dilational interfacial rheological properties were also measured after aging the interface before imposing a periodic oscillation. This aging time allows particles to migrate from the bulk of aqueous suspension to the interface leading to particle concentration and structuration at the interface. The effect of aging time on the interface for suspensions in pure deionized-water is presented in Fig. 5 (f = 0.1 Hz) and Supplementary Figures 9 and 10 (f = 0.2 Hz), and it is almost negligible for the Laponite 0.5 wt.% and 1.0 wt.% suspensions.

For the higher concentration suspension, 1.5%, both the viscous and elastic modulus slightly increases with aging time, but the effect is also very weak. The effect of aging time on the 1.5% Laponite suspension in 0.1 M NaCl solution is significant. The elastic modulus increases, up to approximately 600 mN/m after aging the interface for only 30 minutes.

The addition of sodium chloride to the suspension clearly changes the mechanical behavior of the oil-water interface. The suspension with 1.5 wt % of Laponite and 0.1 M of NaCl presents a very strong elastic behavior, the elastic modulus is higher than 500 mN/m at f = 0.1 Hz after 30 minutes of aging time, which is close to ten times the value of the equilibrium interfacial tension. The results show that the elastic response at higher Laponite concentration is a strong function of the frequency of the imposed oscillation; being higher at the lower frequency. We suggest this can be explained considering that, at low frequency, there is sufficient time to rebuild the interfacial particle structural network within one oscillation cycle, in analogy with bulk rheometric measurements on Laponite^41^. The strong elastic behavior of the interface that we observe is consistent with the work reported by Ashby and Binks^40^, in which emulsions prepared with an aqueous phase containing Laponite 1.5 wt.% are stable in 0.1 M NaCl solution.

Oil-water emulsions prepared with the same liquid systems used here are stable for up 30 days in the cases at which the interface shows elastic behavior, which hinders the drainage between oil drops, avoiding coalescence^41^. Our observations indicate that although the Laponite and salt concentration were so high that a continuous particle structure is not formed in the bulk (the bulk suspension is in the Flocculation region of the phase diagram^1^), a two-dimensional trapped particle network can be formed at the interface, leading to the strong enhancement of the elastic behavior. We attribute this to Laponite flocs being trapped at the drop interfaces, and that the flocs form a connected two dimensional network with voids. We believe that the flocculation (at 0.1 M NaCl concentration) occurs in the bulk prior to capillary trapping of the flocs at the drop interface, which is in accordance with our observed Pickering layer thickness in the μm range (~4 μm). The two-dimensional capillary trapping could modify floc-floc interaction dynamics compared to the bulk situation, as well as possibly deform (“extend” or “flatten”) the individual flocs during and beyond the time needed to form the percolating network. This could possibly reorganize the Pickering layer’s internal clay nano-structure during time, a process that would be linked to the rotational and translational degrees of freedom of the individual Laponite particles, and thus both short term and long term dynamics could very well be different on the Pickering films compared to the bulk case. Future work beyond the present study should thus include linking the Pickering trapping structure and dynamics to translational and rotational degrees of freedom of the particles, such as previously investigated for three dimensional cases^42^,^43^.

The ability to tune the mechanical behavior of interfaces opens the possibility of controlling the dynamics of interface breakup and coalescence during flow, which can lead to more stable emulsions without the use of surfactants, changes in the phase diagram of emulsions and design complex dispersions of soft capsules with elastic shells with different applications such as in biomedical and oil industries.

## Methods

### Experimental

Different Laponite RD^®^ (Rockwood additives) suspensions were used as the aqueous phase, with varying concentrations of clay particles (from 0% to 1.5%) and salinity (0 and 0.1 M NaCl solution). The suspensions were prepared^44^ by first dispersing Laponite in deionized water (0.16 μS/cm at 25 °C) using an IKA WERKE Ultra-Turrax^®^ T25 basic homogeniser (rotor-stator) with a 1.8 cm head operating at 17,500 rpm for 30 min while cooling the sample in an ice/water bath. Laponite-salt (NaCl) suspensions were prepared by adding salt into Laponite RD^®^ dispersions using an ultrasonic bath (40 kHz for at least 40 min). All dispersions were transferred into stoppered glass vessels and were kept refrigerated at 10 °C prior to use.

The oil phase was a paraffinic mineral oil (Drakeol7^®^). To remove the polar and amphiphilic impurities from the industrial grade oil, it was washed several times with excess of deionized water, decanted and centrifuged before been used in the experiments.

The viscosity of the oil and different Laponite suspensions were measured at room temperature by using an Ubbelohde viscometer at ambient temperature (~20–25 °C). The reported results correspond to the average of at least three measurements and are presented in Supplementary Table 1.

As expected, the viscosity of the water phase rises with the addition of the particles, reaching 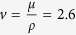 mm^2^/s for the Laponite 1.5 wt% suspension.

As described above, the addition of salt reduces the electrostatic repulsion between the colloidal clay particles, leading to a much higher viscosity (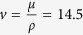 mm^2^/s for the Laponite 1.5 wt%/0.1 M NaCl suspension). It is important to note that all suspensions were prepared in concentrations outside the gelation region^1^.

### Raman spectroscopy

Emulsions of oil in water stabilized by Laponite particles were prepared using a microfluidic device^45^. Samples were placed in a cylindrical custom made sample holder (10 mm diameter and 5 mm height) and sealed with a coverslip. Spectra were acquired with a confocal Raman microscope (alpha-300R, WITec) equipped with a piezo scanner and a high numerical aperture (NA) microscope objective from Nikon. A linear polarized laser was focused with a diffraction-limited spot size and the Raman light was detected by an air-cooled, back-illuminated, electron multiplying EM-CCD camera behind a grating spectrograph. The laser power at the output was approximately 40 mW/cm^2^. Raman spectra were measured using a frequency doubled Nd: YAG green laser (wavelength 532.14 nm, power ~40 mW) and Nikon 20X (NA = 0.40) air objective. The laser light is coupled to the microscope through a single mode optical fiber to avoid irradiating the sample with other frequencies. The grating of the spectrograph used was 1800 grooves/mm and the multi-mode optical fiber used to collect the scattered photons had a diameter of 100 μm. Raman line scans of 10 μm over oil/water interface were performed with a 0.8 μm optical resolution and spectra were taken each 0.1 μm. The excitation light was polarized horizontally in x-direction. Integration time for each spectrum was approximately 5 s, but the spectra are averages of two measurements to improve their quality. Interfaces were assigned by selecting the region between oil and aqueous phases where peak intensity was 75% of maximum intensity. The WITec Project 2.06 software was used for measurement setup and image processing. Chemical Raman images were achieved by using a sum filter, integrating over defined wavenumber areas of the whole spectrum. The filter calculates the intensities within the chosen borders and the background is subtracted by taking the baseline from the first to the second border. The selected sum filters for laponite, oil and water were [658 to 698 cm^−1^], [1414 to 1480 cm^−1^] and [1518 to 1702 cm^−1^] respectively. The scans were performed at approximately 50 μm depth using a 100x Oil immersion objective NA = 1.25.

### Pendant drop method

The interface mechanical behaviour was characterized by the pendant drop method^30^,^34^,^35^,^36^ with a Tracker S tensiometer (Teclis instruments, previously IT Concept, Longessaigne, France) by an axisymmetric drop profile analysis technique. The appropriate aqueous phase (with or without Laponite, with or without salt) was placed in a glass cuvette. An oil droplet was formed at the tip of a J-shaped needle (outer diameter: 1.2 mm) fitted to a glass syringe with total volume of 500 μL. The apparatus is computer controlled, and it allows for time dependent volume deformations of the drop, whilst recording the response of the interfacial traction to the imposed volume (area) deformation. The program WDROP, version T2011, fits the experimental drop profile to the Young–Laplace capillarity equation and provides, as an output, the drop volume *V*, the surface traction γ, and the surface area *A*. All measurements were made at room temperature for an initial drop volume of 22 μL. The steady state interfacial tension was determined by measuring the evolution of the interfacial traction as a function of time for a constant volume drop. Elastic response of the interface was determined by applying a periodic oscillation to the interface. The applied interfacial area oscillations were maintained below amplitude of 10% in order to avoid excessive perturbation of the interfacial layer and breakup of possible particle network structure formed in the interface. Two different measurement frequencies of 0.1 and 0.2 Hz were used, and the oscillations were applied continuously, starting with a freshly formed (0 min), a 10 min and a 30 min aged interface. For one particular case (1.5% Laponite concentration and 0.1 M NaCl solution), the interface aging time was increased to longer time. For each condition, the experiment was stopped when the interfacial traction reached a periodic steady state.

## Additional Information

**How to cite this article**: Gholamipour-Shirazi, A. *et al*. Transition from glass- to gel-like states in clay at a liquid interface. *Sci. Rep.* **6**, 37239; doi: 10.1038/srep37239 (2016).

**Publisher's note:** Springer Nature remains neutral with regard to jurisdictional claims in published maps and institutional affiliations.

## Supplementary Material

Supplementary Information
