# Supplementary material for: Transition from glass- to gel-like states in clay at a liquid interface
Source: Sci Rep. 2016 Nov 24;6:37239. doi: 10.1038/srep37239 (PMC5121648; doi:10.1038/srep37239)
Supplement: Supplementary Information [file srep37239-s1.pdf]

# Supplementary Information

# Transition from glass- to gel-like states in clay at a liquid interface

A. Gholamipour-Shirazi,[a*] M. Carvalho,[a] M. F. G. Huila[b], K. Araki[b], P. Dommersnes[c] and J. O. Fossum[c*]

[a]Department of Mechanical Engineering, Pontificia Universidade Catolica do Rio de Janeiro, Rio de Janeiro, RJ, Brazil.

[b]Institute for Chemistry, Universidade de São Paulo - USP, Sao Paulo, SP, Brazil

[c]Department of Physics, Norwegian University of Science and Technology - NTNU, Trondheim, Norway.

*Corresponding authors email: jon.fossum@ntnu.no

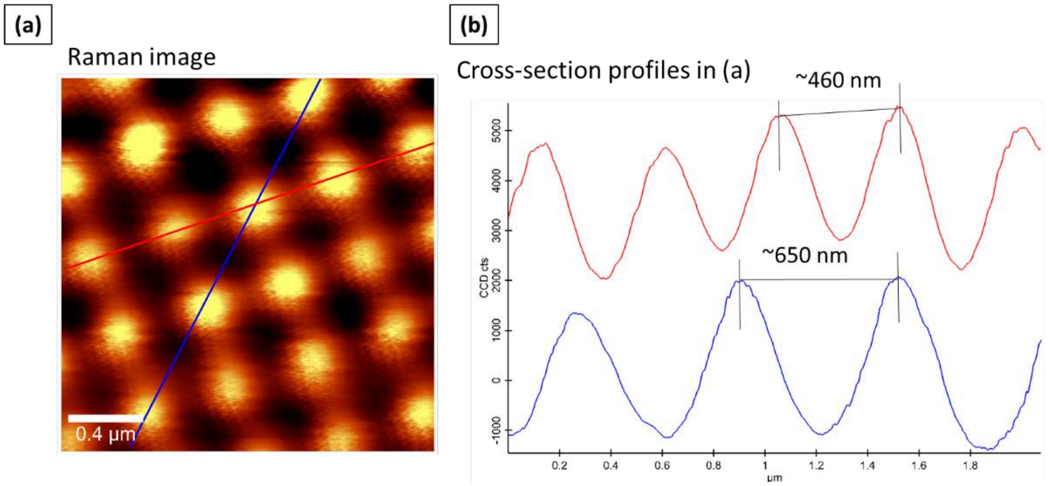


**Supplementary Figure 1.** Confocal Raman imaging obtained by us (Manuel Fernando Gonzalez Huila) of a commercial Ted Pella® 607-STM Waffle Grating Replica for STM calibration. It is a carbon replica with Au/Pd shadowing for X-Y calibration with 2,160 lines/mm crossed lines made on a 400 square mesh, 3 mm copper TEM grid; mounted on a 12 mm stainless steel STM disc. This specimen is a replica of a 2,160 lines/mm waffle pattern diffraction grating. In the top left Raman image, the details of a 4 µm$^2$ area (2.00 µm width x 2.00 µm height) and 400 x 400 pixels, is depicted where the bright regions correspond to the intersections of Au/Pd lines. The cross section line profiles on a line (red) and in diagonal direction (blue) is shown on the right confirming the lateral resolution of about 150 nm. Our Raman images were acquired with a 532 nm laser, 100x air objective, 25 µm pinhole, 1800 g/mm grating and an integration time for each spectrum of 2.7 ms. The image was generated plotting the polarized Rayleigh scattering at 0 cm$^{-1}$ in a perpendicular configuration.

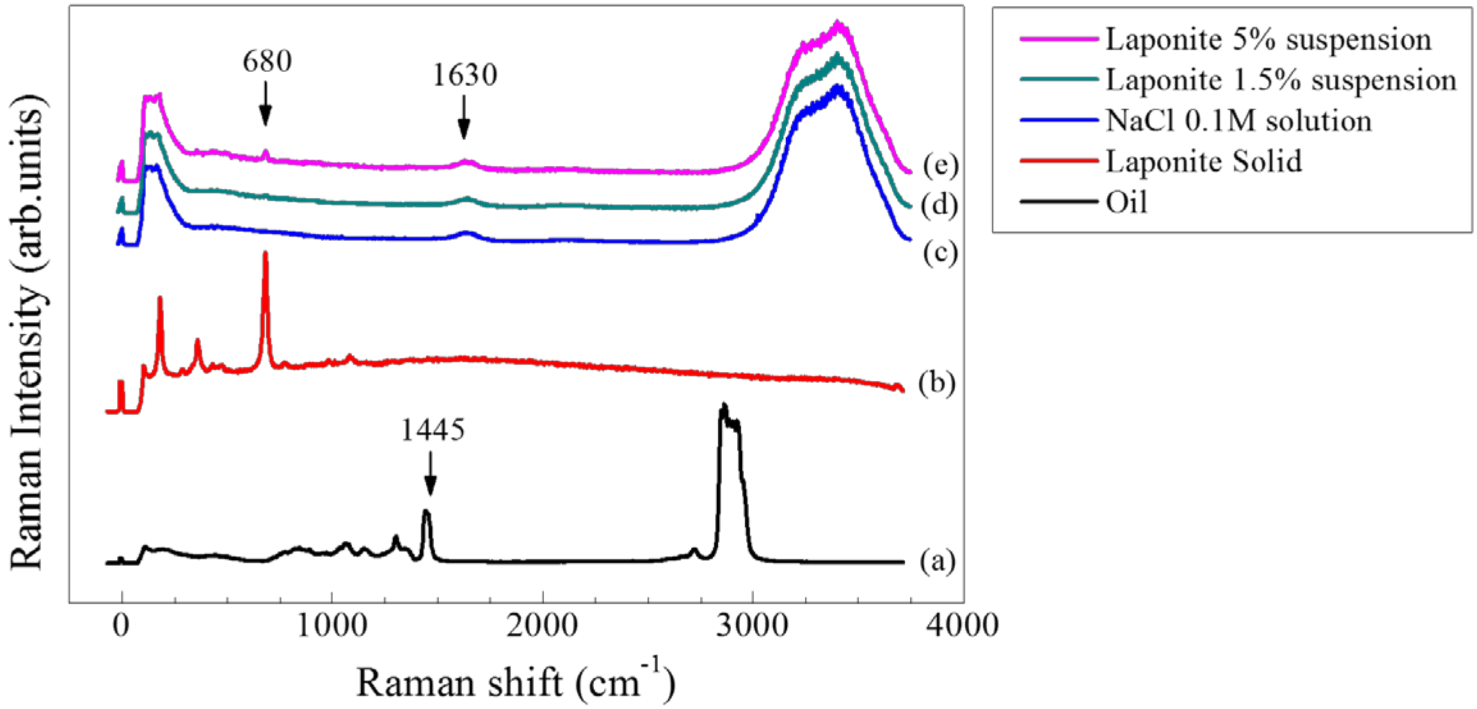


**Supplementary Figure 2.** Raman spectra of employed materials. Raman spectra of mineral oil (a), pure solid laponite (b), a 0.1 M NaCl solution (c), a 1.5 % Laponite suspension (d) and a 5.0 % Laponite suspension (e) in the 0 to 3600 $cm^{-1}$ range (resolution of 3 $cm^{-1}$ using a grating of 600 grooves/mm). Main Raman peaks for laponite, oil and water were pointed at 680, 1445 and 1630 $cm^{-1}$ respectively (corresponding to symmetric Si-O-Si stretching in laponite, $CH_2$ bending in mineral oil and HOH bending in water).

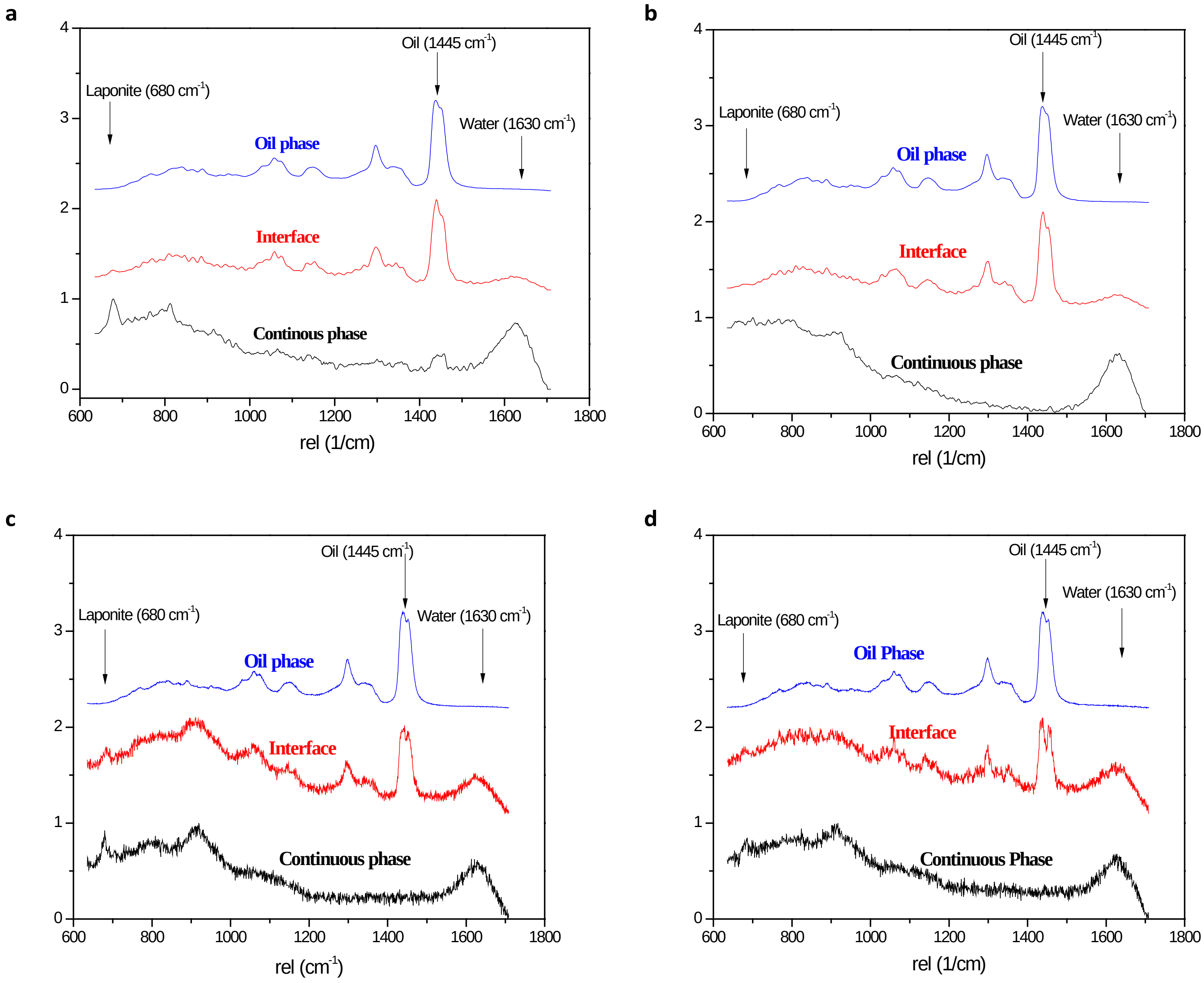


**Supplementary Figure 3:** Raman spectroscopy of a single oil drop in Laponite 1.5wt% dispersion with or without salt. (a) Raman spectra for the oil drop in Laponite 1.5 wt. %, NaCl 0.1M. (c) Raman spectra for the oil drop in diluted Laponite 1.5 wt. %, NaCl 0.1M. It was diluted 1:1 v:v by NaCl 0.1M (d) Raman spectra for the oil drop in Laponite 1.5 wt.% (e) Raman spectra for the oil drop in diluted Laponite 1.5 wt.%. It was diluted 1:1 v:v by deionized water

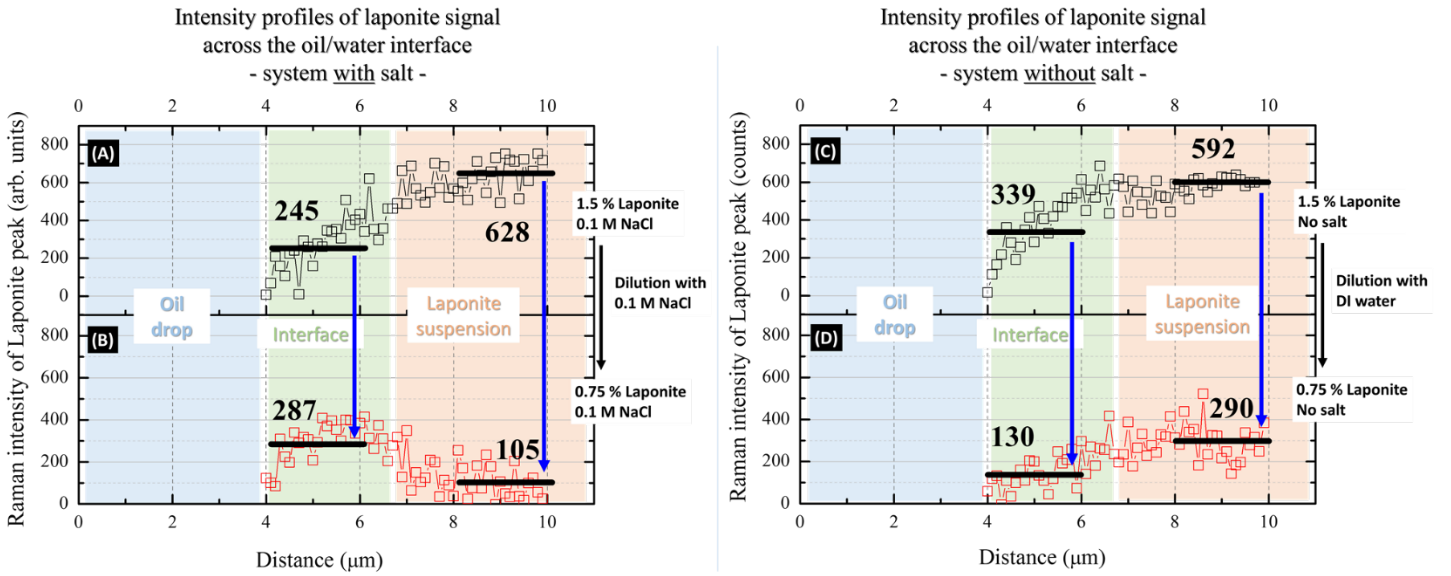


**Supplementary Figure 4:** Raman intensity vs. distance plots monitoring the 680 $cm^{-1}$ laponite peak. Note that the Raman intensities were not normalized. Only Raman peaks with more than 1 count were considered.

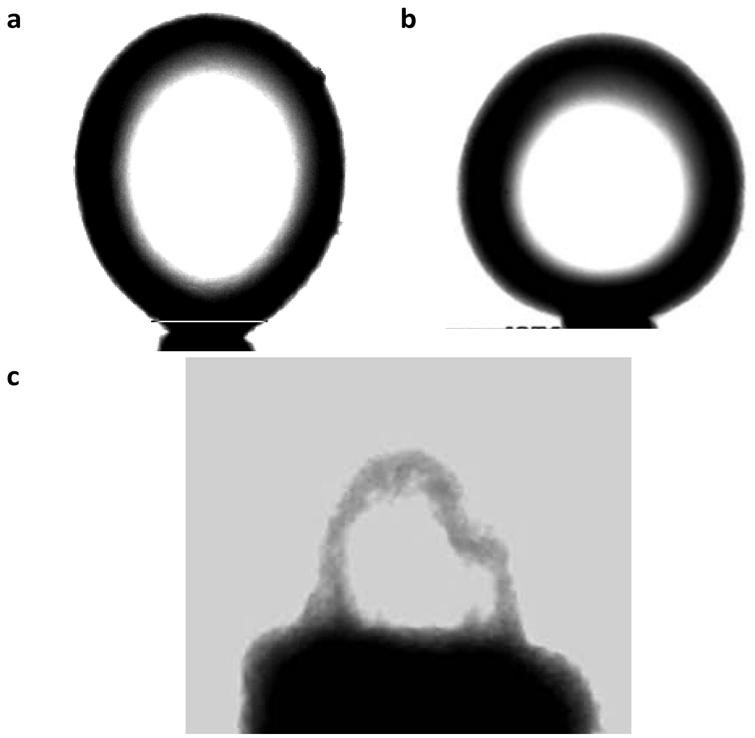


**Supplementary Figure 5:** Effect of aging time on the drop shape.(a) Fresh formed oil drop in the dispersion of Laponite 1.5 wt.% and NaCl 0.1M. (b) The overnight aged oil drop in the dispersion of Laponite 1.5 wt. % and NaCl 0.1M. The drop is spherical. (c) Interface crumpling for an overnight aged drop of oil in the dispersion of Laponite 1.0 wt. % and NaCl 0.1M.

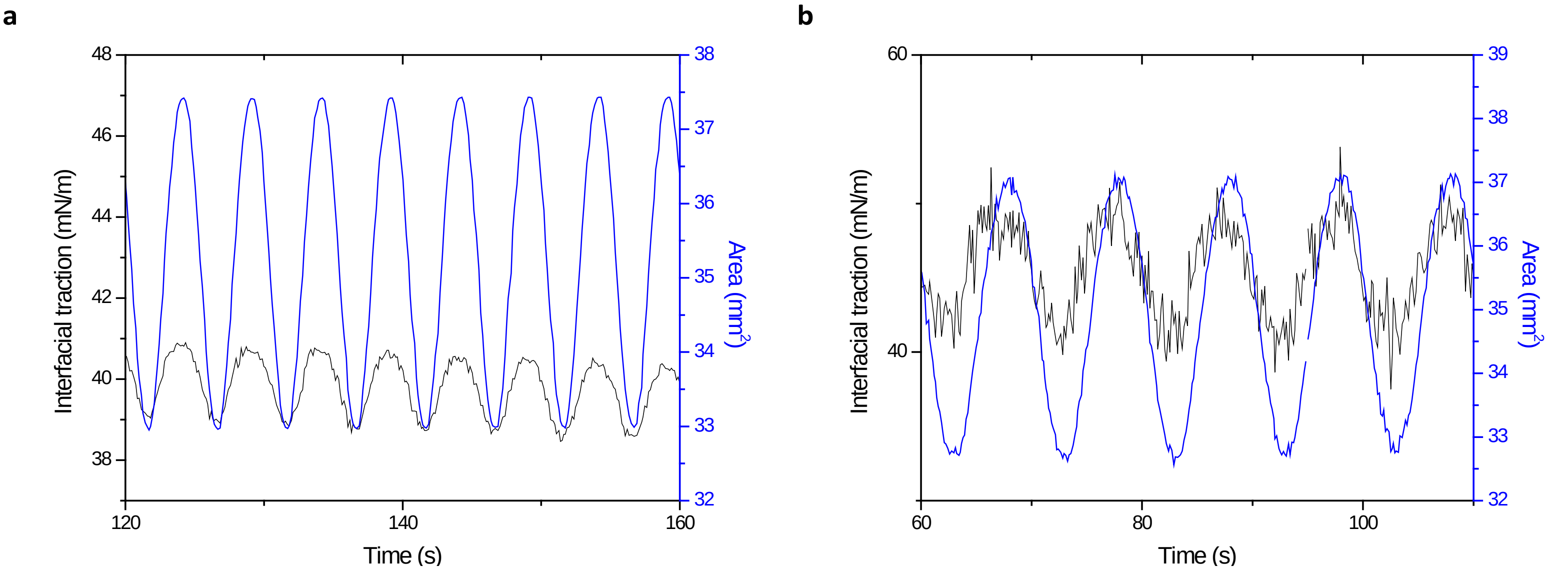


**Supplementary Figure 6:** Evolution of the interfacial traction for an imposed periodic oscillation of surface area. (a) For the oil drop oscillating in deionized water at 0.2Hz. (b) For the oil drop oscillating in Laponite 1.5 wt%, NaCl 0.1M at 0.1 Hz. The volume amplitude for both cases is 2 µL.

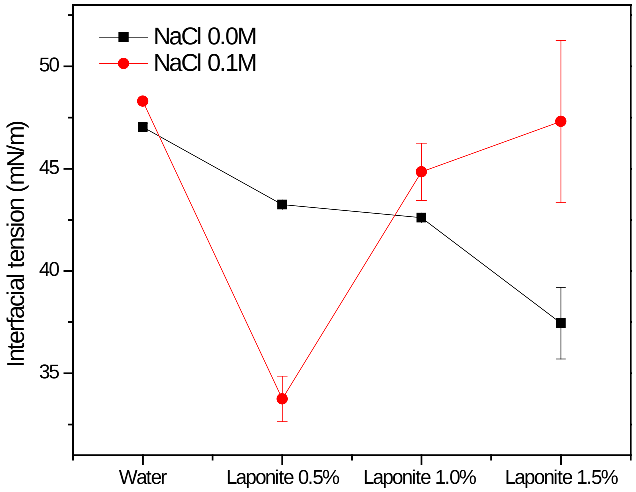


**Supplementary Figure 7:** Steady state values of interfacial tension for different Laponite dispersions in oscillatory tests for the aging time of 0 min. Volume amplitude is 2 µL at the frequency of 0.2 Hz

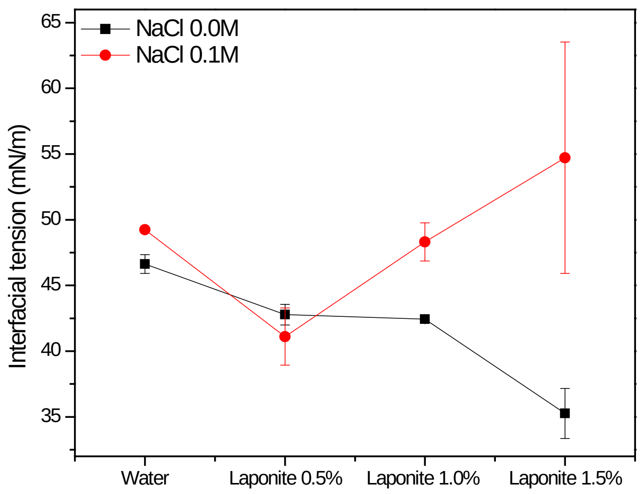


**Supplementary Figure 8.** The steady state values of interfacial tension for different Laponite dispersions in oscillatory tests for the aging time of 0 min. Volume amplitude is 2 µL at the frequency of 0.1 Hz.

a

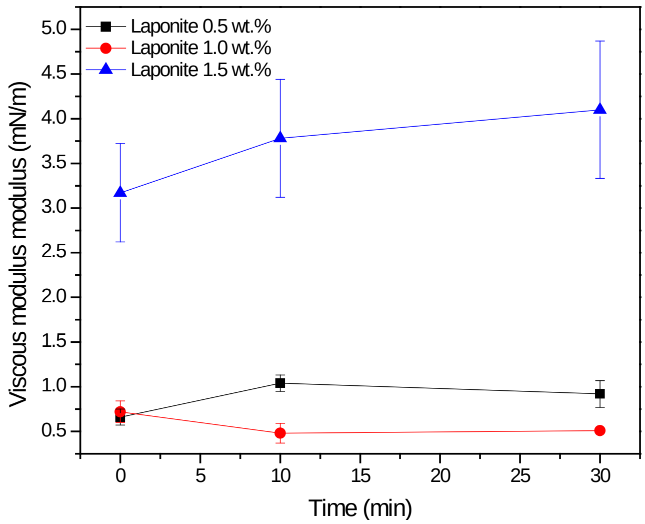


b

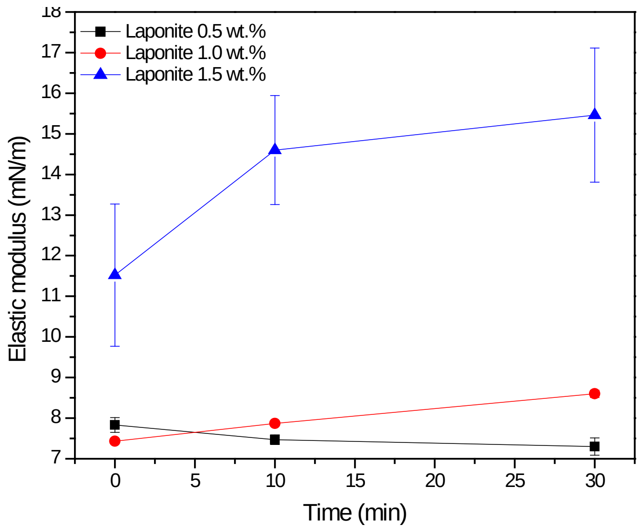


**Supplementary Figure 9:** Effect of aging time at different frequencies on the interfacial viscoelastic moduli of different Laponite dispersions in the absence of salt. Volume amplitude ratio for all cases is 2µL. (a) Viscous modulus at 0.2 Hz (b) Elastic modulus at 0.2Hz

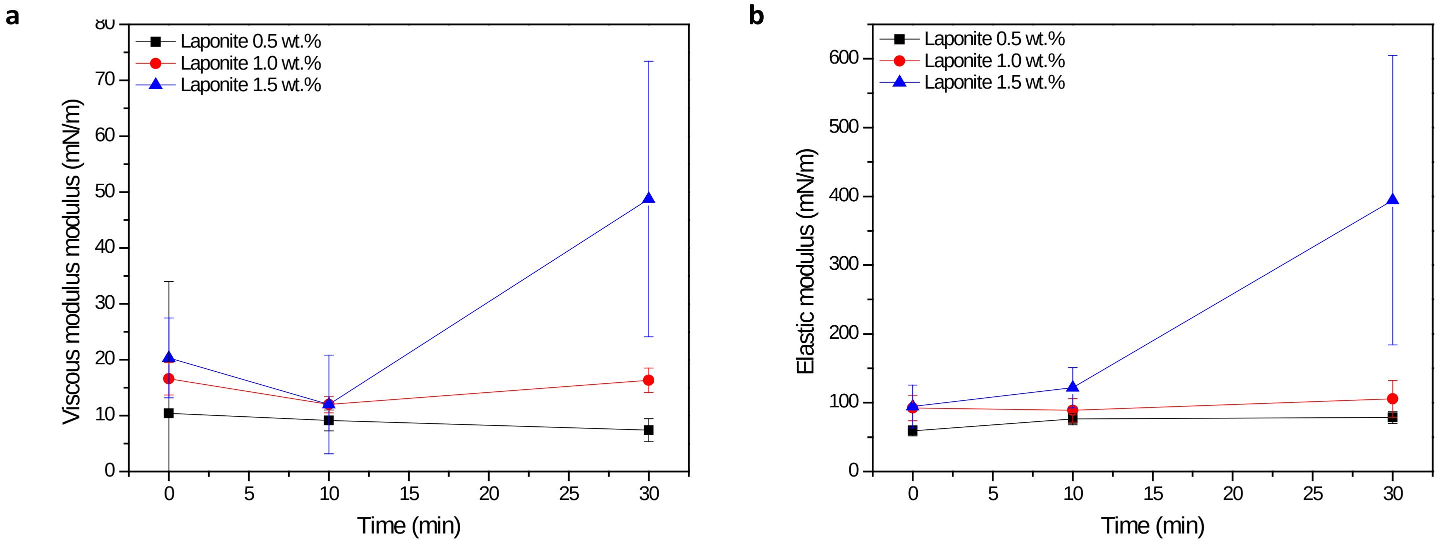


**Supplementary Figure 10:** Effect of aging time at different frequencies on the interfacial viscoelastic moduli of different Laponite dispersions in the presence of salt. Volume amplitude ratio for all cases is 2 µL. (a) Viscous modulus at 0.2 Hz (b) Elastic modulus at 0.2Hz

Supplementary Table 1 Viscosity values for different dispersions

| Fluid | pH | Kinematic Viscosity ($mm^2/s$) |
|---|---|---|
| Oil (purified) | - | 21.69±0.012 |
| Water (deionized) | | 0.92±0.001 |
| Laponite 0.5% | 8.97 | 1.03±0.001 |
| Laponite 0.5%+NaCl 0.1M | 8.95 | 2.60±0.008 |
| Laponite 1.0 % | 9.15 | 2.00±0.003 |
| Laponite 1.0% + NaCl 0.1M | 8.97 | 8.95±1.146 |
| Laponite 1.5% | 9.63 | 2.66±0.008 |
| Laponite 1.5% + NaCl 0.1M | 8.88 | 14.53±1.749 |
